# Temporal Deformation Characteristics of Hydraulic Asphalt Concrete Slope Flow Under Different Test Temperatures

**DOI:** 10.3390/ma18153625

**Published:** 2025-08-01

**Authors:** Xuexu An, Jingjing Li, Zhiyuan Ning

**Affiliations:** 1 School of Railway Engineering, Shaanxi College of Communications Technology, Xi’an 710018, China; lijingjingqi@163.com; 2 Key Laboratory of Eco-Hydraulics in Northwest Arid Region, Xi’an University of Technology, Xi’an 710048, China; ningzhiyuan1108@163.com

**Keywords:** hydraulic asphalt concrete, slope flow, different test temperatures, temporal deformation

## Abstract

To investigate temporal deformation mechanisms of hydraulic asphalt concrete slope flow under evolving temperatures, this study developed a novel temperature-controlled slope flow intelligent test apparatus. Using this apparatus, slope flow tests were conducted at four temperature levels: 20 °C, 35 °C, 50 °C, and 70 °C. By applying nonlinear dynamics theory, the temporal evolution of slope flow deformation and its nonlinear mechanical characteristics under varying temperatures were thoroughly analyzed. Results indicate that the thermal stability of hydraulic asphalt concrete is synergistically governed by the phase-transition behavior between asphalt binder and aggregates. Temporal evolution of slope flow exhibits a distinct three-stage pattern as follows: rapid growth (0~12 h), where sharp temperature rise disrupts the primary skeleton of coarse aggregates; decelerated growth (12~24 h), where an embryonic secondary skeleton forms and progressively resists deformation; stabilization (>24 h), where reorganization of coarse aggregates is completed, establishing structural equilibrium. The thermal stability temperature influence factor (*δ*) shows a nonlinear concave growth trend with increasing test temperature. Dynamically, this process transitions sequentially through critical stability, nonlinear stability, period-doubling oscillatory stability, and unsteady states.

## 1. Introduction

Renowned for exceptional impermeability, superior deformation adaptability, and self-healing capability, hydraulic asphalt concrete (HAC) has emerged as a critical engineering material for impervious structures in pumped-storage power stations [1,2,3]. With increasing implementation of China’s “dual-carbon” strategy, the installed capacity of pumped-storage facilities continues to rapidly expand [4]. However, under harsh service conditions, impervious panels remain persistently exposed to atmospheric elements. During summer, direct solar irradiation elevates surface temperatures to above 70 °C [5,6], inducing asphalt mastic softening and interfacial debonding between aggregates and asphalt binder. This process triggers slope flow deformation [7,8], compromising both long-term panel stability and impermeability, thereby posing significant engineering safety risks.

The high-temperature slope flow behavior of HAC constitutes a viscoplastic deformation process governed by coupled temperature, load, and material properties [9]. Studies reveal a characteristic three-stage deformation evolution: initial linear growth, gradual stabilization, and final steady state, where temperature acts as the primary driver [10]. Critically, Adam et al. [11] identified substantial through-thickness temperature gradients in asphalt panels under solar exposure (surface-to-core differentials ≤ 28 °C). Rapid water-level fluctuations may induce abrupt cooling at 10 °C/min, accumulating significant thermal stresses. Ning et al. [12,13] demonstrated through compression tests that HAC modulus decays exponentially above 40 °C, while shear strength at 60 °C decreases by >60% versus 20 °C. These findings establish a mechanical basis linking temperature-induced property degradation to slope flow mechanisms. Recent studies demonstrate that material modifications (e.g., rubber modified asphalt mixtures enhancing fracture energy by 17.4~21.9% [14]) and mix design parameters including gradation index, asphalt aggregate ratio, filler content, and epoxy modifiers significantly influence deformation resistance [15,16,17]. Meng et al. [18] and Li [19] highlighted the critical role of aggregate skeleton stability (enhanced by higher gradation index) in resisting shear. Cai et al. [20], Zou et al. [21], and Han et al. [10] identified asphalt content as the predominant factor influencing flow value, followed by aggregate and filler contents. Zhang et al. [22,23] further observed a sharp nonlinear flow surge when filler content exceeded 13%, attributable to viscosity reduction. Sun et al. [24] confirmed that epoxy modification significantly enhances HAC’s thermal stability, moisture resistance, and crack resistance. However, emerging evidence indicates that the temporal evolution of HAC deformation under thermal gradients exhibits inherent nonlinearity and state transitions beyond conventional linear viscoelastic frameworks. Logistic models and chaos theory provide rigorous frameworks for interpreting complex damage progression. Ali et al. [25] established a binary logistic model predicting the tertiary stage of permanent deformation in asphalt concrete, demonstrating its capability to capture threshold-dependent phase transitions governed by air voids, gradation, and dynamic modulus. Similarly, Rys et al. [26] applied ordered logistic regression to quantify the odds ratio of low-temperature cracking in high-modulus asphalt concrete, validating its utility for modeling material state transitions under thermal stress. Wu and Fu [27] identified three distinct evolutionary phases in concrete impact response: stable state, periodic state, and chaotic state with chaotic dominance increasing with loading amplitude.

Although existing research effectively characterizes temperature and mix parameter effects on final flow values under static thermal conditions, such studies predominantly employ fixed-temperature tests. This approach inherently neglects the deformation’s complex temporal evolution under dynamic thermal gradients, particularly during critical intermediate stages involving structural reorganization. Coupled viscoelastoplastic phase transitions of asphalt binder and evolving frictional-aggregate skeleton interactions generate strongly nonlinear dynamics inadequately captured by linear models. Nonlinear dynamics and chaos theory provide established frameworks for quantitatively characterizing the evolution of internal status in heterogeneous materials [28]. To address this gap, we developed a temperature-controlled slope flow intelligent test apparatus. Using this apparatus, tests were conducted at four temperatures (20 °C, 35 °C, 50 °C, 70 °C) to capture complete temporal evolution of slope flow. Based on nonlinear dynamics theory, we reveal how thermal stability evolves through critical stability, nonlinear stability, period-doubling oscillatory stability, and unsteady states, providing deeper theoretical insights for preventing deterioration in impervious panels within high-temperature-region pumped-storage plants.

## 2. Test Procedure

### 2.1. Sample Preparation

The HAC specimens consisted of asphalt binder, aggregates, and mineral filler. Karamay 70# petroleum asphalt was selected, with a design asphalt content of 7.0%. The HAC specimens consisted of asphalt binder, aggregates, and mineral filler. All technical properties of the asphalt and aggregates complied with the Chinese standard Test Code for Hydraulic Asphalt Concrete (DL/T 5362-2018) [29]. Based on the Chinese standard Code for Design of Asphalt Concrete Facings and Cores for Embankment Dams (NB/T 11015-2022) [30] and literature [31], the aggregate gradation was determined using the following formula:

(1) $$P_{i}=\alpha +(100-\alpha )\times \frac{{\left(D_{i}\right)}^{m}-{\left(0.075\right)}^{m}}{{\left(D_{\max}\right)}^{m}-{\left(0.075\right)}^{m}}$$

where *P_i_* is the passing rate of sieve size *D_i_* (%), *α* is the filler content (particles ≤ 0.075 mm), *D_max_* is the maximum aggregate size (mm), *D_i_* is the target sieve size (mm), and *m* is the gradation index.

**Figure 1:**
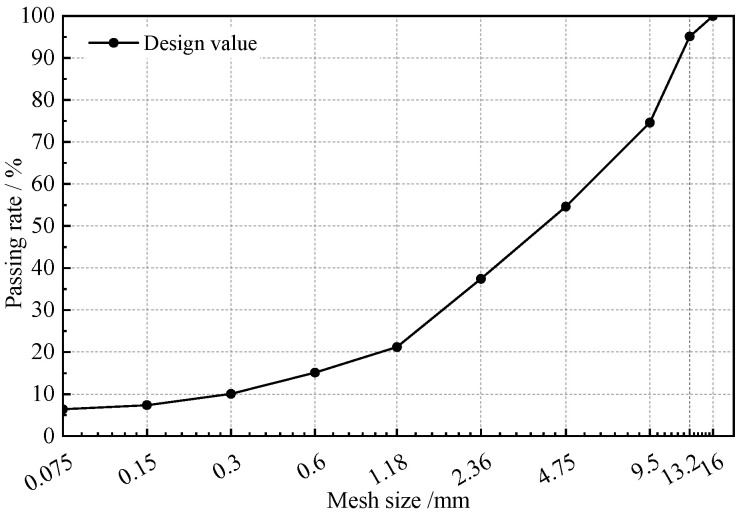
Aggregate gradation curves for asphalt concrete.

A gradation index of *m* = 0.35 was adopted, and the corresponding gradation curve is shown in Figure 1.

Marshall specimens (Φ101.6 mm × H63.5 mm) were fabricated in the laboratory. All specimen preparation followed the requirements specified in the Chinese standard Test Code for Hydraulic Asphalt Concrete (DL/T 5362-2018) [29]. Key steps included: (1) Heating the asphalt mixture to 150 °C, transferring it to a mixing pot, and stirring thoroughly to ensure homogeneity; (2) Transferring a portion of the mixture into a mold and compacting it with a heated spatula using 15 insertions around the perimeter and 10 insertions at the center; (3) Securing the mold in a compaction machine for Marshall compaction; (4) Cooling the specimen and mold naturally to room temperature after compaction, then demolding with a hydraulic ejector; (5) Curing the demolded specimens at room temperature for 24 h before slope flow testing.

**Figure 2:**
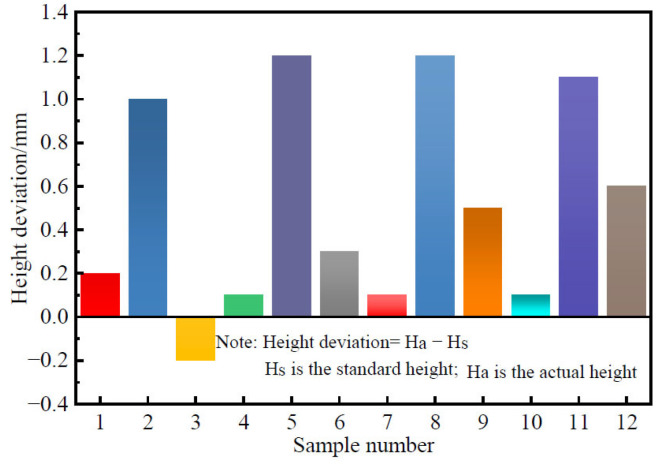
Height deviations in asphalt concrete Marshall specimens.

To minimize the influence of internal defects from Marshall specimen preparation on test results, an RSM-SY5 non-metallic acoustic wave detector was used for acoustic wave testing on all specimens. Specimens exhibiting significant acoustic wave dispersion were discarded. The maximum height deviation among the Marshall specimens was 1.2 mm (Figure 2), below the ±1.3 mm limit specified in the Chinese standard Test Code for Hydraulic Asphalt Concrete (DL/T 5362-2018) [29]. After testing, the average density and porosity of the asphalt concrete Marshall specimens were 2.371 g/cm^3^ and 1.78%, respectively.

### 2.2. Developed Test Apparatus

**Figure 3:**
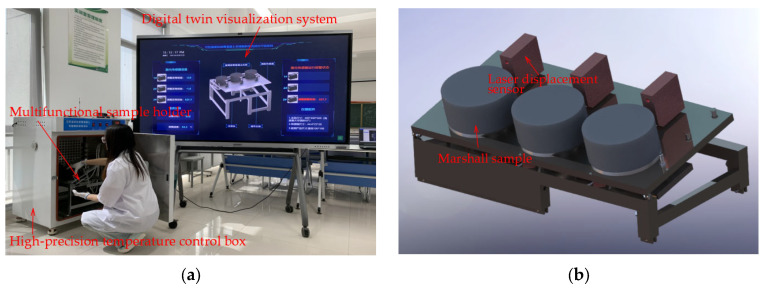
Temperature-controlled slope flow intelligent test apparatus: (**a**) High-precision temperature control box and digital twin visualization system; (**b**) multifunctional sample holder and laser displacement sensor.

As defined in the Chinese standard Code for Design of Asphalt Concrete Facing and Cores for Embankment Dams (NB/T 11015-2022) [30], the slope flow value represents the deformation of hydraulic asphalt concrete measured under standardized conditions of slope gradient, temperature, and duration. The temperature-controlled slope flow intelligent test apparatus (Figure 3) was developed by the authors’ research team specializing in asphalt concrete damage theory and applications. The apparatus comprises the following: (a) a multifunctional sample holder with adjustable slope angle; (b) a high-temperature-resistant laser displacement sensor (accuracy: 0.01 mm); (c) a high-precision temperature control chamber; (d) a wireless data transmission module; and (e) a digital twin visualization system. All laser displacement sensors were laboratory-calibrated prior to testing. Specifically, sensors were calibrated at 70 °C by continuously monitoring a fixed 50 mm distance for 48 h until deviations from the reference distance fell below 0.02 mm. The integrated software system enables real-time remote monitoring of slope flow data and sensor status.

### 2.3. Test Plan

HAC impervious panels in hydropower stations experience significant diurnal and seasonal temperature variations during service. Under direct summer solar irradiation, panel surface temperatures reach 1.6~2.0 times the ambient temperature. Elevated temperatures soften the asphalt binder, causing debonding at aggregate interfaces under combined gravitational load, slope inclination, and panel thickness. This triggers slope flow deformation that severely compromises impermeability. To quantify the ambient temperature (*T*) effects on HAC thermal stability, we implemented the test plan in Table 1. Triplicate tests were performed per condition to ensure reliability.

**Figure 4:**
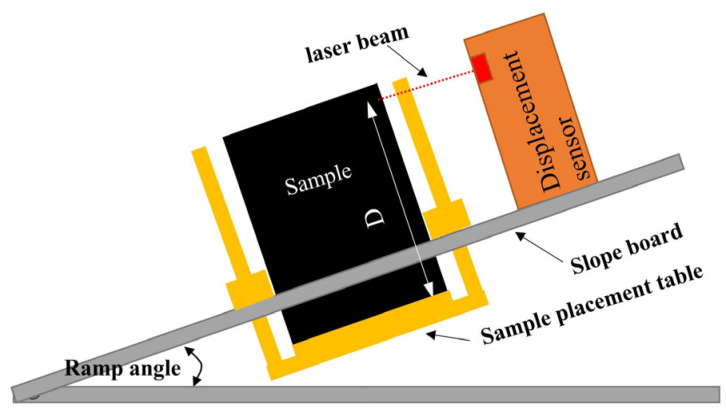
Schematic of slope flow.

(1) Initial setup: According to the test plan, the multifunctional sample holder’s slope was set to 1:1.7, and the temperature inside the high-precision temperature control box was programmed to the target *T*.
(2) Sample installation: Once the temperature inside the high-precision temperature control box stabilized, the box door was opened, and the multifunctional sample holder extracted. High-temperature-resistant adhesive was uniformly applied to the placement table, followed by specimen adhesion.
(3) Laser displacement sensor calibration: The laser displacement sensor was activated and calibrated by adjusting the reference slope board until the beam aligned with the predefined *D* = 50 mm point from the specimen base (Figure 4).
(4) Test initiation: The multifunctional sample holder with the installed specimen was smoothly pushed back into the high-precision temperature control box, and its door was closed. The laser displacement sensor readings were zeroed before initiating 48 h isothermal testing.
(5) Data acquisition: Real-time slope flow values were recorded and downloaded via the digital twin system throughout testing.

## 3. Result Analysis

### 3.1. Temporal Evolution of Slope Flow Value

**Figure 5:**
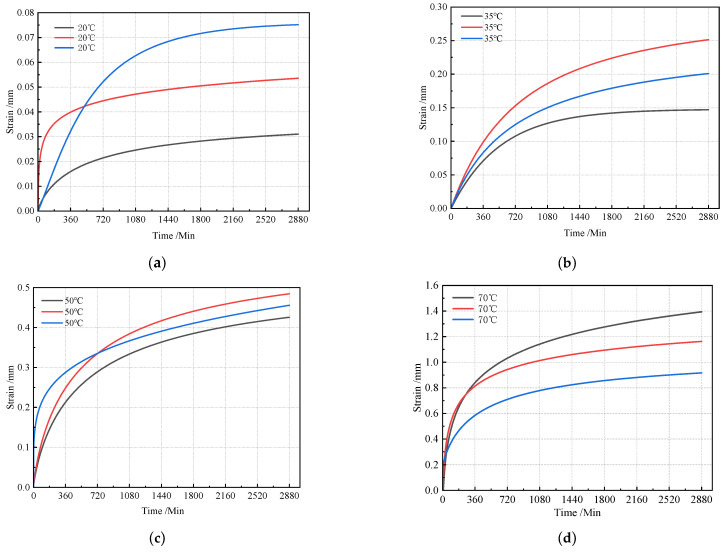
Temporal evolution of slope flow at different temperatures: (**a**) 20 °C; (**b**) 35 °C; (**c**) 50 °C; (**d**) 70 °C.

The asphalt mastic—a blend of asphalt binder and filler—plays a critical role in the thermal stability of HAC. This stability is governed by both the cohesive strength of the asphalt mastic and frictional resistance among aggregate particles. Notably, the cohesion exhibited by the asphalt mastic is highly temperature-dependent. To elucidate the impact of test temperature on HAC thermal stability, we measured time-dependent slope flow values under four temperatures (20 °C, 35 °C, 50 °C, and 70 °C), as depicted in Figure 5.

Analysis of Figure 5 reveals that slope flow evolution follows a consistent temporal pattern across all tested temperatures. The slope flow initially increases rapidly, then gradually stabilizes over the test duration. Specifically, within 0~12 h (720 min), flow increases at a high rate, reflected by steeper initial slopes. Subsequently, during 12~24 h (720~1440 min), the increase rate progressively diminishes, evidenced by decreasing tangent slopes. Finally, during 24~48 h (1440~2880 min), HAC specimens reach a quasi-steady state, with negligible changes in slope flow until test termination.

**Figure 6:**
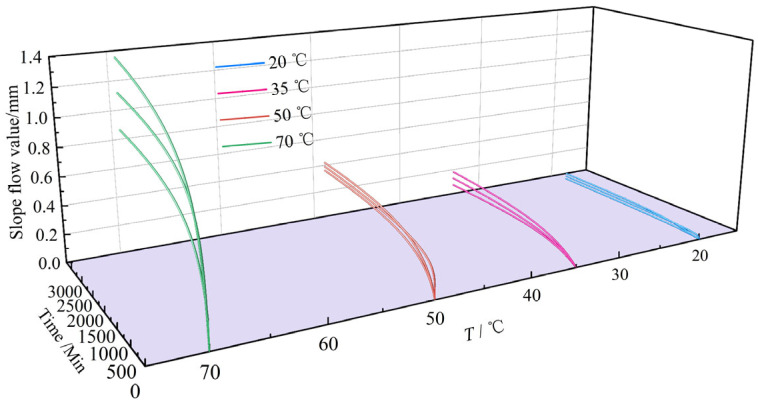
Slope flow value curves of asphalt concrete across test temperature.

**Figure 7:**
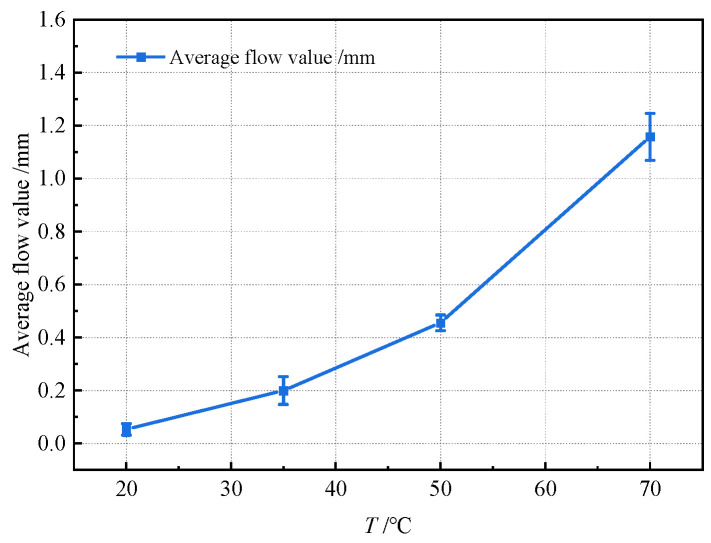
Relationship between average slope flow value and test temperature (*T*).

Collectively, these results demonstrate the temporal evolution of slope flow in asphalt concrete across test temperatures. To quantify temperature effects on thermal stability, Figure 6 summarizes slope flow curves for each temperature, while Figure 7 plots the average flow value (defined as the mean of triplicate 48 h measurements) versus test temperature. In Figure 7, error bars denote the maximum absolute deviation from the mean across triplicate samples per temperature.

Figure 6 and Figure 7 reveal a distinct nonlinear increase in slope flow with rising temperature. The flow increment is modest at low temperatures but accelerates substantially at temperatures above 35 °C. This behavior stems from the combined effects of gravitational force and thermal softening of the asphalt mastic. Elevated temperatures enhance the lubricating effect of softened mastic, facilitating greater deformation under gravity and consequently higher slope flow values.

### 3.2. Temporal Evolution of Slope Flow Completion Rate

**Figure 8:**
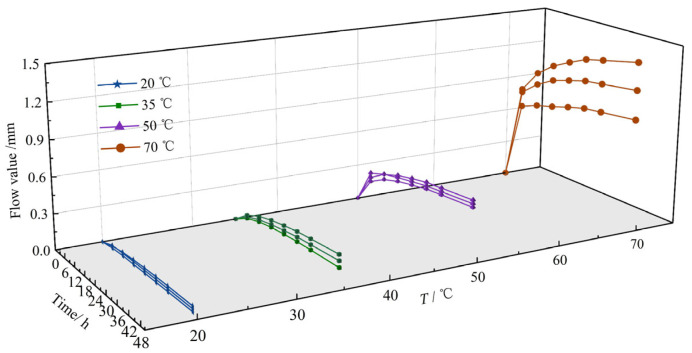
Slope flow evolution at key time for all test temperatures (*T*).

**Figure 9:**
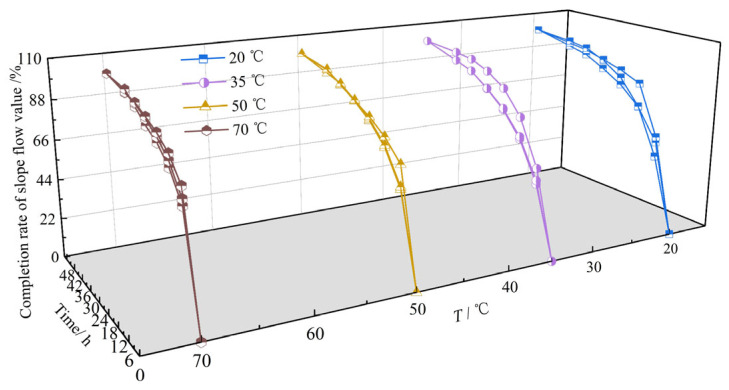
Temporal evolution of slope flow completion rate under different temperatures (*T*).

To gain deeper insight into deformation progression, Figure 8 presents slope flow evolution curves at key times (6, 12, 18, 24, 30, 36, and 48 h) for all test temperatures. Figure 9 shows the corresponding temporal evolution of slope flow completion rate (defined as the ratio of instantaneous to final (48 h) slope flow) across temperatures.

Analysis of Figure 8 and Figure 9 indicates that within the initial 6 h, slope flow value typically exceeds 50% of the final value. During 6~12 h, an additional 20% deformation accumulates, resulting in 50~70% slope flow completion rate. This rapid initial deformation stems from HAC’s composition: designed for impermeability, it contains elevated proportions of fine aggregate, filler, and asphalt binder. Although enhancing pore-filling and impermeability, this composition increases initial mobility under thermal and mechanical loading. From 12 to 24 h, deformation attains 70–90% of the final completion rate, with a markedly reduced growth rate. This deceleration occurs due to sufficient softening of the asphalt mastic, which reduces the mixture’s viscosity. Here, under combined thermal and gravitational effects, coarse aggregates rearrange to gradually form a stable secondary skeleton. Finally, during 24~48 h, slope flow changes become negligible, indicating stabilization. This stability arises from the fully formed coarse aggregate skeleton, which effectively resists further deformation and enhances slope thermal stability.

### 3.3. Stage Characterization of Slope Flow Evolution

The analysis in Section 3.2 demonstrates three consistent stages in HAC slope flow evolution under varying temperatures: rapid growth (Stage I), decelerated growth (Stage II), and stabilization (Stage III). This staged behavior is quantitatively validated through slope flow completion rate. As Table 2 summarizes, statistical analysis identifies objective inflection points at 12 h and 24 h, defining the following phase boundaries: Stage I (0~12 h): The completion rate surges from 0% to 70.3% (70.31 ± 4.98% at 12 h), reflecting rapid deformation accumulation. Stage II (12~24 h): Growth decelerates markedly, with completion rate reaching 87.7% (87.69 ± 3.46% at 24 h). Stage III (24~48 h): Deformation stabilizes, with completion rate attaining 100% (100 ± 0.0% at 48 h), confirming asymptotic convergence.

This behavior is fundamentally linked to temperature-dependent phase changes in the asphalt binder. Asphalt exhibits (i) brittle behavior below its glass transition temperature, (ii) viscoelasticity between glass transition temperature and softening point, and (iii) pronounced viscoplasticity above the softening point. Consequently, increasing test temperature enhances the deformation capacity of asphalt mastic. The original coarse aggregate skeleton is disrupted. Under gravitational forces, aggregates reorganize into a stable secondary structure. Upon completed reorganization, significant further slope flow increase ceases. The deformation mechanism evolves through the following three stages:

Stage I (0~6 h): At test initiation, the pre-heated oven temperature exceeds the specimen’s initial temperature. Sudden thermal exposure rapidly elevates HAC temperature. As temperature rises, asphalt mastic softens, significantly enhancing its deformation capacity. Under gravity, the initially stable skeleton progressively destabilizes from the surface inward. Concurrently, a secondary structure begins to form. During this phase, slope stability is governed by interactions between the primary and emerging secondary structures. Slope flow exhibits an approximately linear time-dependence.

Stage II (12~24 h): As testing progresses, the primary structure progressively disintegrates while the secondary structure matures. The slope flow value growth rate diminishes markedly due to the nascent secondary skeleton developing sufficient mechanical resistance to counteract deformation forces from combined thermal softening and gravitational loading. Consequently, flow resistance increases, inducing a sharp decline in deformation rate. The completion rate typically reaches 70~90% within this stage.

Stage III (24~48 h): At this stage, the primary structure collapses completely, and a stable secondary skeleton is established. The mixture exhibits significantly enhanced slope thermal stability, evidenced by a plateau in slope flow value with negligible further increase (<1%). The completion rate reaches 90~100%, confirming a structurally equilibrated state that resists additional flow under sustained thermal loading.

## 4. Nonlinear Mechanical Behavior Characteristics of HAC Thermal Stability

### 4.1. Thermal Stability Temperature Influence Factor

To quantify the temperature sensitivity of asphalt concrete mechanical properties, researchers [32,33] have established the strength temperature influence factor. Building on prior research, we define the thermal stability temperature influence factor (*δ*) as follows:

(2) $$\delta =\frac{U_{T}}{U_{70}}$$

where *δ* represents the ratio of slope flow value at various test temperature conditions to its slope flow value at 70 °C; *U*_T_ is the slope flow value (mm) at test temperature *T*; and *U*_70_ denotes the value at 70 °C, both measured at a fixed slope ratio of 1:1.7 over 48 h.

**Figure 10:**
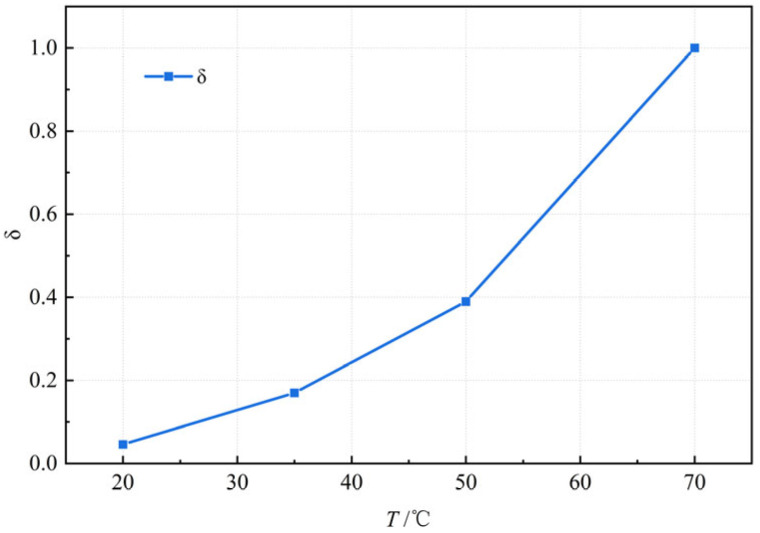
Relationship between thermal stability temperature influence factor (*δ*) and the test temperature (*T*).

Substituting the test data into Equation (2) yields the relationship curve between the thermal stability temperature influence factor and the test temperature, as shown in Figure 10.

Figure 10 reveals a three-stage growth pattern for *δ* with increasing temperature: (i) slow growth (<35 °C), (ii) accelerated growth (35~50 °C), and (iii) rapid growth (>50 °C). The overall response exhibits a pronounced concave nonlinearity. This nonlinearity originates from the strong temperature dependence of the asphalt mastic’s mechanical properties. Beyond a critical temperature threshold, additional heating progressively diminishes the asphalt mastic’s solid-like mechanical response. Consequently, *δ* asymptotically approaches a limit rather than increasing indefinitely. Therefore, the evolution of *δ* with temperature is well characterized by a logistic model:

(3) $$\delta =\frac{\beta }{1+e^{-\lambda U^{*}+\psi }}$$

(4) $$U^{*}=U-U_{70}$$

where *β*, *λ*, and *ψ* are fitting coefficients: *β* denotes the upper limit of *δ*, *λ* is the promotion coefficient, U* represents the equivalent test temperature, *U* represents the test temperature (*T*), and *U*_70_ is the reference temperature (70 °C).

**Figure 11:**
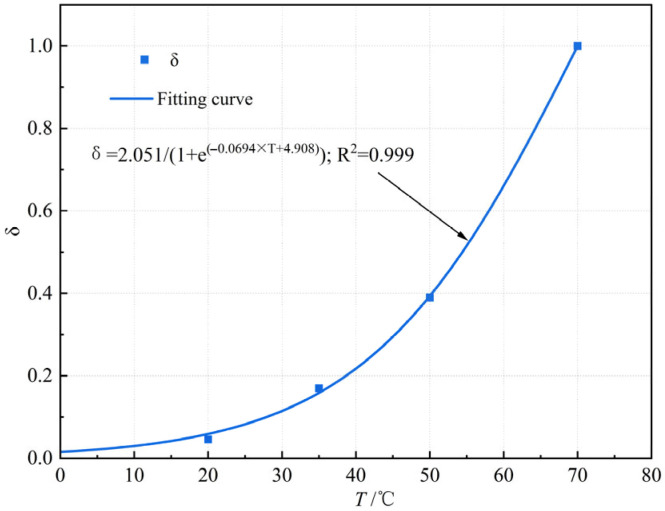
Fitting curve of thermal stability temperature influencing factor (*δ*) versus test temperature (*T*).

Based on Equations (3) and (4), the data in Figure 10 were fitted using MATLAB R2014b software, resulting in the fitted curve of thermal stability temperature influence factor versus test temperature (Figure 11).

Figure 11 indicates that the logistic mathematical model achieves a correlation coefficient of fit greater than 0.9, confirming its suitability for characterizing test temperature on the thermal stability influence factor.

### 4.2. Disorder Characteristics of the Thermal Stability Temperature Influence Factor Sequence

To further investigate the nonlinear mechanical behavior characteristics of thermal stability temperature influence factor sequence during temperature evolution, the aforementioned logistic mathematical model is transformed based on nonlinear dynamics and chaos theory.

Based on Equation (3), a dimensionless parameter *φ* = *β*/*δ* is defined, where *φ* represents the ratio of instantaneous thermal stability temperature influence factor to its limiting value. This yields the following equation:

(5) $$\varphi =\frac{\beta }{\delta }=\frac{1}{1+e^{-\lambda U^{*}+\psi }}$$

Differentiating Equation (5) yields the following:

(6) $$\frac{d\varphi }{dU^{*}}=\lambda \frac{1}{1+e^{-\lambda U^{*}+\psi }}(1-\frac{1}{1+e^{-\lambda U^{*}+\psi }})=\lambda \varphi (1-\varphi )$$

This derivation leads to the one-dimensional mapping equation:

(7) $$\varphi_{n+1}=\lambda \varphi_{n}(1-\varphi_{n})$$

where *φ_n_* represents the internal state of asphalt concrete at a certain equivalent temperature U*, and *n* denotes a dimensionless number for different internal states of asphalt concrete.

**Figure 12:**
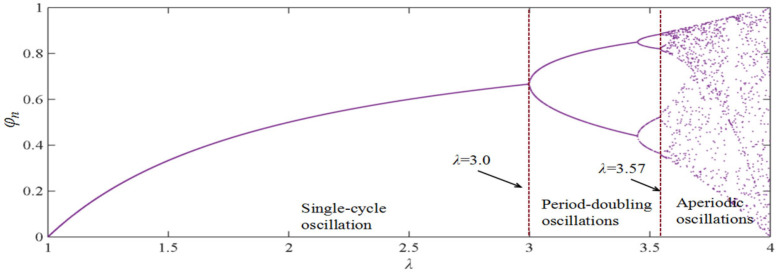
The variation characteristics of *φ_n_* with the thermal stability temperature influence factor promotion coefficient *λ.*

Research on the logistic equation has demonstrated [34] (Figure 12) the following:

When the thermal stability temperature influence factor promotion coefficient *λ* satisfies 1.0 < *λ* < 3.0, *φ_n_* converges to a non-zero stable value through iterative computation. This behavior reflects regular oscillations in temperature-induced micro-deformations, indicating a structurally stable system.

When 3.0 ≤ *λ* < 3.57, period-doubling oscillations emerge in *φ_n_* after successive iterations. This indicates declining system stability, during which enhanced asphalt mastic fluidity facilitates coarse aggregate reorganization into secondary structures.

When λ ≥ 3.57, aperiodic oscillations develop in *φ_n_* during iteration. This signifies pronounced temperature dependence of thermal stability, revealing instability in coarse aggregates during secondary skeleton formation.

**Figure 13:**
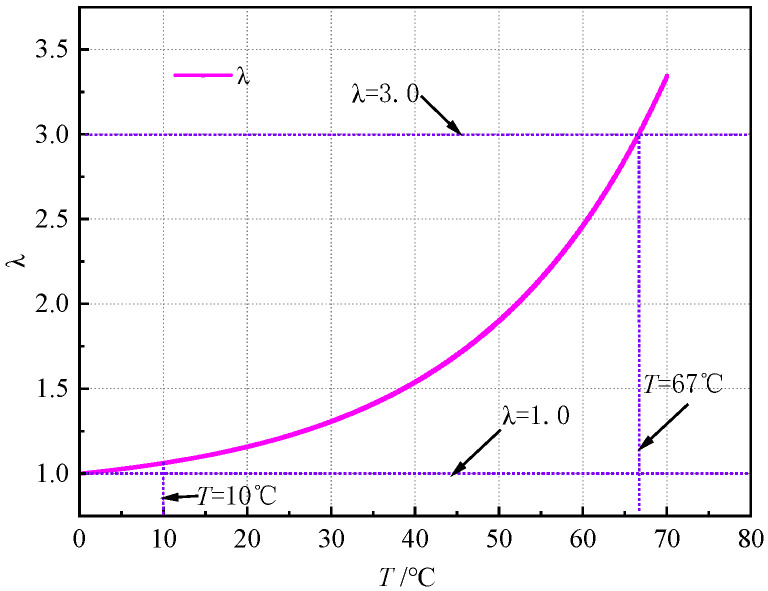
One-dimensional mapping curve of thermal stability temperature influence factor promotion coefficient (*λ*) versus test temperature (*T*).

Substituting experimental data into Equation (7) yields the one-dimensional mapping curve of *λ* versus test temperature in Figure 13.

Figure 13 reveals three distinct behavioral regimes:

Regime I (*T* < 10 °C): As test temperature increases, *λ* remains stable near 1.0 with negligible growth, indicating minimal structural reorganization. In this regime, the asphalt mastic exhibits predominantly elastic behavior, yielding negligible slope flow deformation.

Regime II (10 °C ≤ *T* ≤ 67 °C): λ increases continuously from 1.0 to 3.0 with rising test temperature, reflecting progressive softening of the asphalt mastic. Consequently, the internal system transitions to a nonlinearly stable state, where temperature-induced microstructural adjustments (e.g., aggregate reorientation) produce oscillatory but predictable slope flow deformation.

Regime III (*T* > 67 °C): When temperature exceeds the asphalt mastic’s softening point, λ increases rapidly from 3.0, surpassing 3.57 with further heating. Here, the internal system enters a non-periodic oscillation state, and the heightened fluidity of the asphalt mastic prevents effective aggregate interlocking, leading to significant slope flow deformation.

## 5. Discussion

The findings of this study provide critical insights into the temporal deformation mechanisms of HAC under varying temperatures, with direct implications for improving dam design and slope stability in pumped-storage power stations. The identified three-stage deformation pattern (rapid growth, decelerated growth, and stabilization) reflects the evolution law of slope flow under a high-temperature environment. Specifically, the rapid growth stage (0~12 h) highlights the vulnerability of HAC to sudden temperature rises, which disrupt the primary aggregate skeleton. This suggests that dam design in high-temperature regions should incorporate measures to reduce surface temperature fluctuations, such as reflective coatings or shading systems, to delay the onset of deformation. Furthermore, the nonlinear growth of the thermal stability temperature influence factor (*δ*) with increasing temperature emphasizes the need for material optimization. For instance, using asphalt binders with higher softening points or adjusting gradation indices to enhance aggregate skeleton stability can significantly improve resistance to slope flow. The study’s logistic model for *δ* evolution offers a predictive tool for engineers to assess thermal stability under projected climate conditions, aiding in the selection of appropriate materials and design parameters.

## 6. Conclusions

- The slope flow deformation of asphalt concrete exhibits a distinct three-stage temporal evolution pattern: (1) Rapid growth stage (0~12 h): The slope flow value increases rapidly, reaching 50~70% of the final deformation. This is attributed to thermal disruption of the primary skeleton structure formed by coarse aggregates. (2) Decelerated growth stage (12~24 h): The growth rate decreases sharply, with deformation attaining 70~90% of the final value. During this stage, an embryonic secondary skeleton forms and progressively develops deformation resistance. (3) Stabilization stage (24~48 h): Deformation stabilizes (>90% completion) as coarse aggregates complete reorganization, establishing a stable secondary skeleton that effectively resists further flow.
- The thermal stability of asphalt concrete is synergistically controlled by the phase-transition behavior of the asphalt binder and the reorganization of coarse aggregates. At elevated temperatures, the asphalt phase transition dominates initial deformation, while coarse aggregate reorganization governs stabilization by forming a secondary skeleton in later stages. When the test temperature exceeds the asphalt’s softening point, the internal system enters an aperiodic oscillatory state. This systematic disordering prevents coarse aggregates from establishing an effective secondary skeleton.
- Beyond the asphalt’s softening point, non-periodic instability compromises aggregate interlocking, accelerating deformation. This necessitates thermal mitigation strategies (e.g., reflective surface coatings) and material optimization (e.g., high-softening-point binders, optimized gradation indices) for slope integrity in pumped-storage facilities.

## Figures and Tables

**Table 1:** Test plan for HAC slope flow.

No.	Variable Name	Variable Value	Other Variables	Number of Samples
1	Test temperature (*T*)	20 °C	Tamp angle *α* = 30°	3
2		35 °C		3
3		50 °C		3
4		70 °C		3

**Table 2:** Mean and standard deviation of slope flow completion rate versus test time.

Test Time/h	6	12	18	24	30	36	48
Average value/%	52.95	70.305	81.4825	87.68833	92.96917	96.115	100
Standard deviation/%	4.66	4.98	4.61	3.46	2.30	1.63	0

## Data Availability

The original contributions presented in this study are included in the article. Further inquiries can be directed to the corresponding authors.
